# Identification of Plastics in Mixtures and Blends through Pyrolysis-Gas Chromatography/Mass Spectrometry

**DOI:** 10.3390/polym16010071

**Published:** 2023-12-26

**Authors:** Chiara Gnoffo, Alberto Frache

**Affiliations:** Department of Applied Science and Technology, Politecnico di Torino, V.le Teresa Michel, 5, 15121 Alessandria, Italy; chiara.gnoffo@polito.it

**Keywords:** polymers, py-GC/MS, blends, calibration curves

## Abstract

In this paper, the possibility of detecting polymers in plastic mixtures and extruded blends has been investigated. Pyrolysis–gas chromatography/mass spectrometry (py-GC/MS) allows researchers to identify multicomponent mixtures and low amounts of polymers without high spatial resolution, background noise and constituents mix interfering, as with molecular spectrometry techniques normally used for this purpose, such as Fourier transform infrared spectroscopy (FTIR) and Raman spectroscopy and differential scanning calorimetry (DSC). In total, 15 solid mixtures of low-density polyethylene (LDPE), polypropylene (PP), polystyrene (PS), polyamide (PA) and polycarbonate (PC) in various combinations have been qualitatively analyzed after choosing their characteristic pyrolysis products and each polymer has been detected in every mix; thus, in extruded blends of high-density polyethylene (HDPE), PP and PS had varying weight percentages of the individual constituents ranging from 10 up to 90. Moreover, quantitative analysis of these polymers has been achieved in every blend with a trend that can be considered linear with coefficients of determination higher than 0.9, even though the limits of quantification are lower with respect to the ones reported in the literature, probably due to the extrusion process.

## 1. Introduction

According to the United Nations’ estimates, the world population will reach 10 billion by 2050 [1] and, consequently, the demand for consumer goods, such as plastic, will rise. This material has been largely used for decades now due to several characteristics which makes it resistant to compression, traction, impact, corrosion and rigid but cheap and lightweight at the same time [2,3]. The most produced polymers in the world are mainly polyethylene (PE) and polypropylene (PP), followed by poly(vinyl chloride) (PVC), polystyrene (PS) and poly(ethylene terephthalate), as well as polyamide (PA), and polycarbonate (PC), and their production is going to increase, as well as the pollution and waste coming from this source [4,5,6].

The issues related to plastic’s impact on several ecosystems, such as in marine and soil environments, have been largely investigated, since 90% of plastic products are not recycled [7,8]. Regardless of plastic’s resistance to degradation, different environmental mechanisms can lead to plastic deterioration, such as photodegradation as a consequence of solar irradiation, thermal degradation due to high temperatures, mechanical degradation as a result of the action of external forces, and biotic degradation caused by organisms [9,10,11], while in aqueous media abrasion, photolysis and biotic lead to plastic fragmentation [12,13,14]. This results in the formation of micro- and nanoplastics (MPs and NPs), characterized, respectively, by the largest dimension ranging from 1000 µm up to 1 µm or by at least one dimension restricted to the nanoscale [15,16], although pieces with a size lower than 5 mm are currently regarded as microplastics [17].

The magnitude of MPs’ and NPs’ effects on sea is huge [18,19,20]: 80% of total plastic waste is caused by land-based sources, degrades into smaller pieces and can be ingested by aquatic organisms, which can also get tangled up in sea-based waste such as fishing gears [21,22]. The consequences concern the health of both marine wildlife and human beings since plastic enters the food chain through fishing [23,24,25].

Similarly, MPs’ contamination in the land is constantly increasing due to sewage, landfill and agricultural waste [26,27,28], with effects on soil organisms and biota [29,30] and resulting bioaccumulation through human alimentation [31,32]. In particular, farm activities consumed 7.4 million tons of plastics in 2019 [33], partly related to mulching; its advantages concern cultivation quality and yield, as well as a reduction in insect infestation and irradiation exposure [34]. Mulch films are mostly made of low-density polyethylene (LDPE) and recently of biodegradable materials as well, such as polybutylene adipate terephthalate (PBAT), poly(lactic acid) (PLA) [35,36,37] and starch-based biodegradable plastics [38], since MPs coming from traditional polymers can lead to quality soil alteration, organic matter impoverishment caused by C/N metabolism speed-up and release of greenhouse gases (GHG) [39].

One of the main issues is related to MPs’ characterization and to their size, as their toxicity is strongly connected to their dimension [40,41,42]. Several methods can detect MPs and NPs; regarding chemical composition and non-destructive analysis, two of the most widely used techniques to characterize MPs are Fourier transform infrared spectroscopy (FTIR) and Raman spectroscopy, which are molecular spectroscopy technologies based, respectively, on the absolute frequencies at which a sample absorbs infrared radiation and the relative frequencies at which a sample scatters radiation [9]. FTIR analysis also showed promising results with aged mulching films [43], while Raman mapping has been able to simultaneously visualize and identify five MPs [44]. However, there are certain limits which make them difficult to use in some cases [45,46]. Raman spectroscopy requires minimal sample preparation, does not interfere with water, and succeeds in detecting MPs smaller than 1 µm [47], but it suffers from background noise, soil components and organic matter, such as additives, colors and contaminants [44], solved by using, for example, time-of-flight secondary ion mass spectrometry (ToF-SIMS) [48]. The limitations of FTIR are related to its spatial resolution, ranging from 10 up to 20 µm [49]; micro-FTIR (µ-FTIR) can detect up to 5 µm, but it requires contact between the probe and the sample, which can be damaged in the process [47].

Among destructive techniques, differential scanning calorimetry (DSC) and thermogravimetric analysis (TGA)-based methods, such as TGA-DSC, TGA-FTIR, TGA-MS, are the most common, as well as thermal extraction desorption–gas chromatography–mass spectrometry (TED-GC-MS) and pyrolysis–gas chromatography–mass spectrometry (py-GC/MS). DSC analysis can identify and quantify single polymers, but exhibits some limits when dealing with multicomponent mixtures [50]; as concerns the TGA technique, there is an overlapping of the temperature range of decomposition between different polymers [51].

Mass spectrometry analysis techniques provide complementary solutions to identify single polymers and to detect them in mixtures and blends, which is the aim of this study by using py-GC/MS. The operation of this technique is widely known in the literature [52,53,54]; it is characterized by a pyrolizer, which initially degrades large molecules into smaller ones in an inert atmosphere (in this case, helium) and it generates volatile degradation of characteristic products, which will be separated into a GC column made of copper with a stationary phase made of silica. This will lead to a fingerprint of the products, the chromatogram. Later, the spectrometric detection results in a mass spectrum, typical of the volatile residues caused by MS [55]. This technique is able to boost the sensitivity of the analysis, even when dealing with samples from complex environmental matrixes, and it can be combined with other methods, such as atomic force microscopy-based infrared spectroscopy (AFM-IR) [56] or µ-FTIR [57] in order to gather insights about micro- and nanoplastics’ size and shape, since this information is lost during pyrolysis along with number of particles [58] due to the fact that this characterization method is destructive and may alter the chemistry of the samples [59].

La Nasa et al. [60] highlighted the issues and the recent progresses in the quali-quantitative determination of microplastics via py-GC/MS since several studies have also investigated the likelihood of identifying single polymers when they are UV irradiated [11], but there is lack of unique and specific procedures. Picò et al. [59] demonstrated the potentiality of this technique not only with MPs, but also with organic matter from the environment and this can lead to analysis of potential pollutants for soil due to the presence of plastic [61,62]. Regarding composites, wood–plastic composite products have been studied so as to assess the interaction with the matrix, either high-density polyethylene (HDPE) [63] and PLA [64], or with a catalyst, for example, ZSM-5 zeolite [65]. Depolymerization was identified as the main thermal degradation process when py-GC/MS was used to investigate LiClO4/poly(vinyl alcohol) PVA composites [66] and it was possible to characterize the pyrolysis products of composite polytetrafluoroethylene (PTFE)/poly(ethylene glycol) (PEO) coatings on aluminum [67] and of glass-fiber-reinforced thermoplastic resin [68], as well as for PLA/acrylonitrile-butadiene-styrene copolymer (ABS) [69] and PE in biodegradable polymer blends [70]. As concerns mixtures, it has been possible to identify eleven types of polymers in a mixture combining two solutions with nine polymers and a solid mixture of two polymers with an inorganic diluent in order to achieve small sample amounts [71]; the identification of different polymers in a mixture can also be achieved through an algorithm, with the lowest detectable amount being equal to 1 µg [72]. In addition, Lou et al. [73] displayed the enhancement in quantification through the study of several typical characteristic peaks of polymers after pyrolysis.

In the literature, several databases collecting single-polymer pyrograms have been collected for qualitative analysis of plastics [54] and linear calibration curves have been collected for mixtures of microplastics in environmental matrix [74]. The purpose of this study is to assess whether it is possible to qualitatively detect single plastics as LDPE, PP, PS, PA and PC in different solid mixtures in order to obtain a database for quantitative analysis of extruded blends of HDPE, PP and PS with varying weight percentages of the individual constituents, as a starting point for further analysis on samples from real case studies.

## 2. Materials and Methods

### 2.1. Materials

Five different pristine polymers were analyzed: Lupolen 1800S LDPE and Purell HP371P, PP supplied by LyondellBasell (Ferrara, Italy), Makrolon 2458 PC supplied by Covestro (Filago, Italy), 3630 PS supplied by Total (Bruxelles, Belgium) and PA Radilon^®^ S HS 105 M NT supplied by RadiciGroup High Performance Polymers (Bergamo, Italy). In order to obtain the blends, high-density polyethylene (HDPE) HB33531 Evalene produced by JG Summit petrochemical corporation (Pasig City, Philippines), PP Moplen HP500N produced by LyondellBasell ((Ferrara, Italy),), and PS Crystal 1810 produced by Total Energies((Bruxelles, Belgium) have been used.

### 2.2. Method

#### 2.2.1. Solid Mixtures and Blends Preparation

In order to obtain 15 solid mixtures, 0.6 mg of each polymer was weighed with an analytical scale with precision of ±0.1 mg and solid-state mixed into DMI sample inserts with a volume equal to 30 µL, according to Table 1, and the weighting step was repeated three times for each system for higher precision. Since the largest particle size of each polymer is lower than 0.5 mm, these mixtures can be considered as being constituted by microplastics, according to their definition [17].

Xplore MC 15 HT twin-screw micro compounder was used for the extrusion process. The temperature was set as 190 °C for all the prepared blends, while the speeds of the screws during the material and the process phase of the material were equal to 50 rpm and 100 rpm, respectively, with a mixing time amounting to 3 min.

Several blends, reported in Table 2, were obtained on the basis of different materials weight percentages; 0.6 mg of each blend were tested.

#### 2.2.2. Characterization Techniques

For chromatographic analysis, GC/MS QP2010 SE supplied by Shimadzu (Tokyo, Japan), coupled with high-performance multimode inlet OPTIC-4, was used. The parameters of the pyrolyzer, GC and MS are reported in Table 3. The GC column is made of fused silica and the stationary phase is dimethyl polysiloxane, a non-polar phase, and its length, interior diameter and film thickness are 30 m, 0.25 mm and 0.5 µm, respectively. In order to prepare the sample for pyrolysis, DMI sample inserts with a volume equal to 30 µL were used. For the pyrolysis step, two ramps were employed; since this method is intended to be used for real samples from environmental matrixes, double-shot pyrolysis allows us to identify molecules that pyrolyze at lower temperatures with respect to the one of the polymers, such as additives, solvents and soil residual [75,76].

The approach for evaluating the LOD (limit of detection) and LOQ (limit of quantification) depends on the peak area of the characteristic pyrolysis product and can be based on its signal-to noise ratio (S/N) [77] or on the standard deviation of the response and the slope [78]. In the first case, an S/N ratio equal to 3 is acceptable for assessing the detection limit, while the quantification can be performed when this value is at least 10. In the latter case, when a calibration of the analyte is made, the following equations are used:

LOD = 3.3s/a(1)

LOQ = 10s/a(2)

A standard deviation (SD) of the peak area of the intercept and a slope of the calibration curve are assumed.

## 3. Results and Discussion

### 3.1. Qualitative Analysis of Mixtures

Among the papers on analysis of mixtures of dissolved polymers, Fisher et al. identified characteristic markers that allow the identification of PE, PP, PS, PA and PC when TMAH is added before pyrolysis on the basis of abundance of polymer products [79]. The choice fell on n-alkanes, n-alkenes and n-alkadienes for PE, 2,4-dimethyl-1-heptene for PP, styrene for PS, ε-caprolactam for PA and bisphenol A for PC, as shown in Table 4; similarly, Matsueda et al. opted for ε-caprolactam and bisphenol A when the polymers are dissolved in an inorganic solvent [71], along with Matsui when treating real microplastic samples from oceanic water [72]. These peaks, along with the highest ones for LDPE, have been chosen for each polymer and in order to validate their identification, the S/N ratio has been evaluated.

Table 5 shows peaks’ recognition of the different types of blends that have been tested. The characteristic pyrolysis products of PP, PS, PA and PC are always detected, with the S/N ratio being greater than 3. Regarding LDPE, instead, only the central peak of triplet groups is recognized, while the molecule with lower retention time and two degrees of unsaturation suffers from interaction with pyrolysis products of other plastics. The peak of the saturated compound can be detected only when PP is not present in the mixtures; otherwise, decane interacts with 2-methyl-3-methylene-nonane from PP pyrolysis.

**Figure 1:**
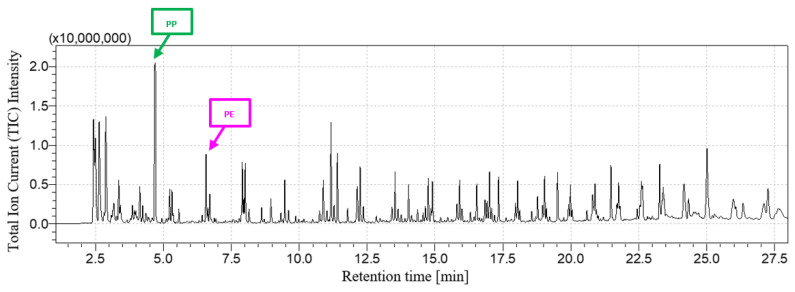
LDPE + PP chromatogram.

**Figure 2:**
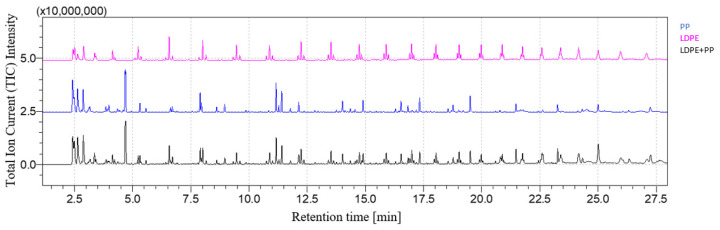
LDPE + PP chromatogram comparison.

Figure 1 and Figure 2 show, respectively, the LDPE + PP chromatogram and its comparison with the LDPE and PP chromatograms. At a retention time equal to 4.74 min, the peak related to 2,4-dimethyl-1-heptene, the main pyrolysis product of PP, is detected, as well as 1-decene after 6.61 min associated with LDPE. As highlighted by Figure 2, the resulting chromatogram is the combination between the chromatograms of LDPE and PP taken individually.

**Figure 3:**
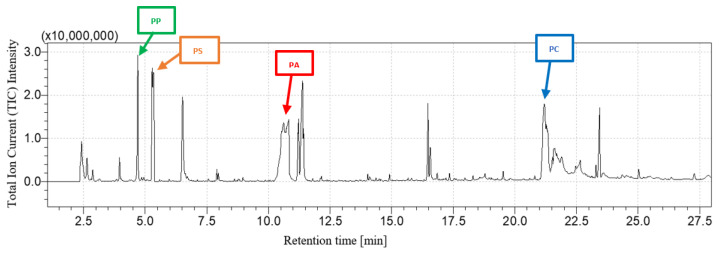
PP + PS + PA + PC chromatogram.

**Figure 4:**
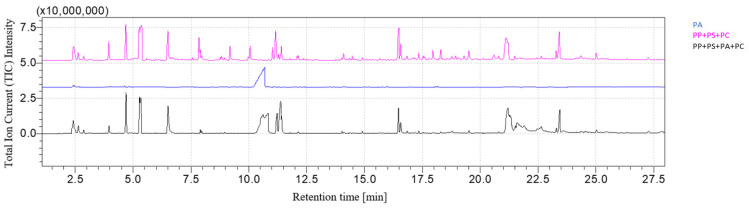
PP + PS + PA + PC chromatogram comparison.

Figure 3 and Figure 4 show, respectively, the PP + PS + PA + PC chromatogram and its comparison with the PP + PS + PA and PC chromatograms. The fingerprints of each constituent of the mixture can be recognized: after pyrolysis, the peaks related to 2,4-dimethyl-1-heptene for PP, styrene for PS, ε-caprolactam for PA and 2,2-bis(4′-methoxylphenyl)propane for PC are detected after 4.74 min, 5.38 min, 10.80 min and 21.2 min, respectively. The rising chromatogram depends on the chromatograms of each polymer, whether they are taken singularly or in mixtures, as shown in Figure 4.

**Figure 5:**
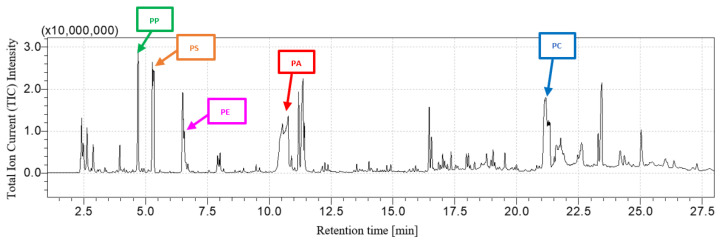
LDPE + PP + PS + PA + PC chromatogram.

**Figure 6:**
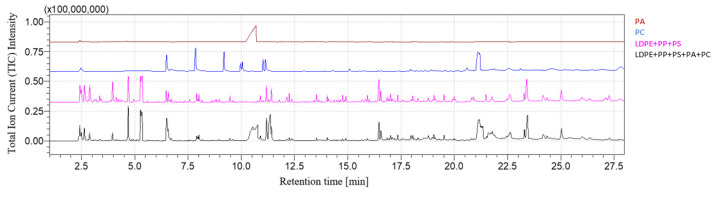
LDPE + PP + PS + PA + PC chromatogram comparison.

Figure 5 and Figure 6 show, respectively, the LDPE + PP + PS + PA + PC chromatogram and its comparison with the LDPE + PP + PS + PA and PC chromatograms. As shown in Figure 5, the reference pyrolysis products of each polymer can be identified without overlapping between peaks related to LDPE (1-decene with retention time equal to 6.61 min), PP (2,4-dimethyl-1-heptene with retention time equal to 4.74), PS (styrene with retention time equal to 5.38 min), PS (ε-caprolactam with retention time equal to 10.80 min) and PC (2,2-bis(4′-methoxylphenyl)propane with retention time equal to 21.2 min). The resultant chromatogram is the set of any polymer or mixture, as highlighted in Figure 6.

All other mixtures’ chromatograms and their comparison are reported in the Supplementary Materials (Figures S1–S29). In all cases, the benchmarks for each polymer, as reported in Table 4, are clearly recognized.

### 3.2. Quantitative Analysis of Blends

In order to quantify the plastics in the blends, two peaks for each polymer have been chosen so as to enhance the accuracy of the quantification. For the determination of peaks area, total ion current (TIC) intensity was evaluated, as already carried out by other authors [80,81]. Table 6 shows the characteristic pyrolysis products for quantitative identification in extruded blends.

**Figure 7:**
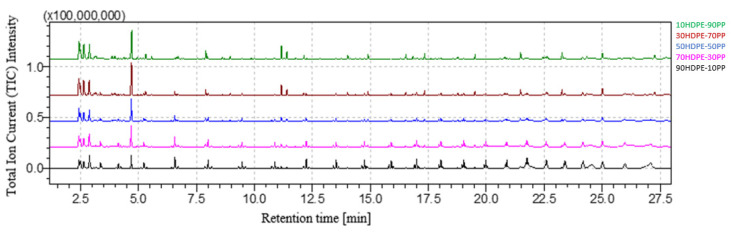
HDPE-PP blends’ chromatograms comparison.

Figure 7 shows the comparison between the chromatograms of 90HDPE-10PP, 70HDPE-30PP, 50HDPE-50PP, 30HDPE-70PP and 10HDPE-90PP. As expected, PP characteristic peaks become higher with increasing weight percentage of PP in the blend, while the heights of the HDPE peaks diminish. Qualitative assessment is always possible for both HDPE and PP.

**Figure 8:**
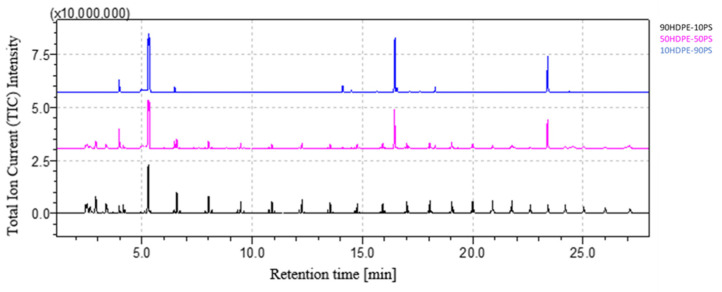
HDPE-PS blends’ chromatograms comparison.

Figure 8 shows the comparison between the chromatograms of 90HDPE-10PS, 50HDPE-50PS and 10HDPE-90PS. As expected, PS peaks’ areas increase with higher weight percentage of PS in the blend, while the heights of the HDPE peaks diminish. Qualitative analysis is always possible for both HDPE and PS.

**Figure 9:**
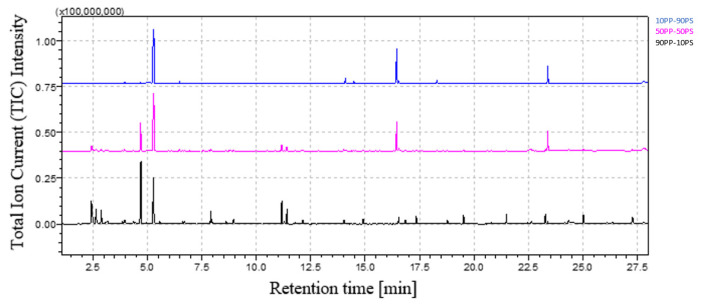
PP-PS blends’ chromatograms comparison.

Figure 9 shows the comparison between the chromatograms of 90PP-10PS, 50PP-50PS and 10PP-90PS. As expected, bibenzyl and 2,5-diphenyl, 1,5-hexadiene peaks’ areas increase with higher weight percentage of PS in the blend, while the heights of the PP peaks diminish. Qualitative assessment is always possible with any weight percentage of PP and PS.

**Figure 10:**
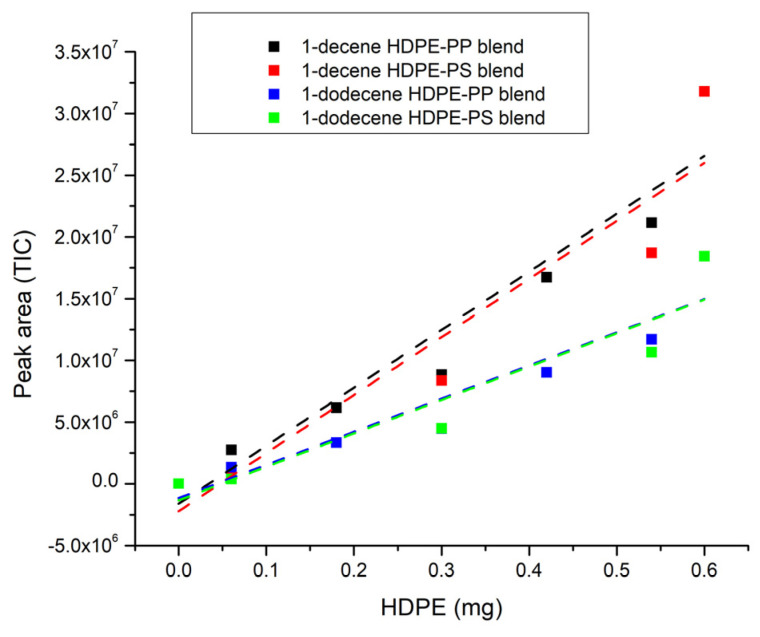
HDPE blends’ trendline (dotted line).

**Figure 11:**
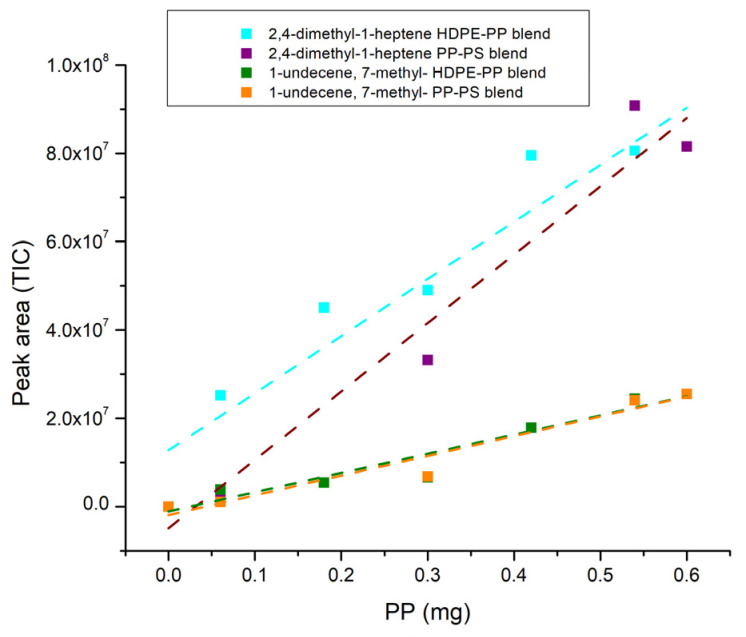
PP blends’ trendline (dotted line).

**Figure 12:**
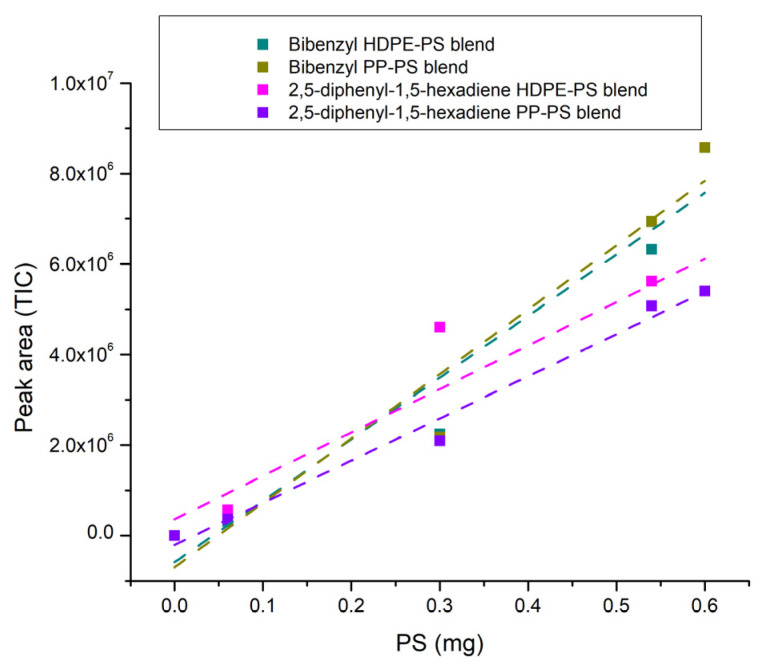
PS blends’ trendline (dotted line).

After collecting the experimental data on blends, the peak area of the characteristic markers for HDPE, PP and PS is reported as a function of percentages by weight transformed into milligrams of the blend’s constituents (Figure 10, Figure 11 and Figure 12) so as to establish the correlation between them and to calculate the LOD and LOQ for each polymer starting from the standard deviation of the intercept and the value of the slope of each calibration curve. The values of the peak areas of 1-decene, 1-dodecene, 2,4-dimethyl-1-heptene, 7-methyl-1-undecene, bibenzyl and 2,5-diphenyl-1,5-hexadiene in different types of blends are reported in the Supplementary Materials (Tables S1–S3).

The trends can be considered linear with R^2^ higher than 0.86, as shown in Table 7. Table 8 displays the values of LOD and LOQ for each polymer in different blends and it can be highlighted that the limit of detection for the pyrolysis products generally ranges from 0.14 mg up to 0.26 mg, except for 2,5-diphenyl-1,5-hexadiene in PP-PS blend, which decreases to 0.09 mg and, as a result, the limits of quantification in this case also differ from the values of the polymers in other blends, which vary from 0.42 mg up to 0.79 mg. These limits are greater than the ones related to non-extruded polymers, whose LOD is usually below 1 μg [78,82,83]; this could be due to the fact that the polymers, either alone or in blends, are subjected to the extrusion process and consequently to a thermal treatment that could lead to morphological modifications [84,85,86] and interactions between constituents in the melting state, and thus to a reduced ability for the detector to identify the polymer characteristic peaks.

## 4. Conclusions

In this work, the possibility of detecting plastics in both solid mixtures and blends has been investigated. Regarding mixtures, the peaks related to the characteristic pyrolysis products of PS, PP, PA and PC are always detectable with an S/N ratio higher than 3, as well as the central peak of the triplet set typical of LDPE. When PP is not present in the mixture, the saturated compound of polyethylene is also identifiable, while the molecule with two degrees of unsaturation interacts with the decomposition products of other polymers.

As concerns extruded HDPE-PP, HDPE-PS and PP-PS blends with varying weight percentage of these polymers, the markers of HDPE, PP and PS are always detectable, except for bibenzyl in the 90HDPE-10PS blend. The limits of detection range from 0.14 mg up to 0.26 mg, and, consequently, it is only possible to make a quantification from a weight percentage equal to 77 for HDPE and PS and 72 for PP. The differences with respect to the limits reported in the literature regarding the order of micrograms are probably due to the extrusion and the interactions between macromolecules of different polymers during the blend processing, whose impacts greatly the microstructure and, consequently, the characteristics of the materials.

## Figures and Tables

**Table 1:** Solid mixtures.

	LDPE	PP	PS	PA	PC
LDPE + PP	0.6 ± 0.1 mg	0.6 ± 0.1 mg	-	-	-
LDPE + PS	0.6 ± 0.1 mg	-	0.6 ± 0.1 mg	-	-
PP + PS	-	0.6 ± 0.1 mg	0.6 ± 0.1 mg	-	-
PS + PA	-	-	0.6 ± 0.1 mg	0.6 ± 0.1 mg	-
PP + PS + PC	-	0.6 ± 0.1 mg	0.6 ± 0.1 mg	-	0.6 ± 0.1 mg
LDPE + PP + PS	0.6 ± 0.1 mg	0.6 ± 0.1 mg	0.6 ± 0.1 mg	-	-
LDPE + PA + PC	0.6 ± 0.1 mg	-	-	0.6 ± 0.1 mg	0.6 ± 0.1 mg
LDPE + PS + PA	0.6 ± 0.1 mg	-	0.6 ± 0.1 mg	0.6 ± 0.1 mg	-
LDPE + PS + PC	0.6 ± 0.1 mg	-	0.6 ± 0.1 mg	-	0.6 ± 0.1 mg
LDPE + PP + PS + PA	0.6 ± 0.1 mg	0.6 ± 0.1 mg	0.6 ± 0.1 mg	0.6 ± 0.1 mg	-
LDPE + PP + PA + PC	0.6 ± 0.1 mg	0.6 ± 0.1 mg	-	0.6 ± 0.1 mg	0.6 ± 0.1 mg
LDPE + PP + PS + PC	0.6 ± 0.1 mg	0.6 ± 0.1 mg	0.6 ± 0.1 mg	-	0.6 ± 0.1 mg
LDPE + PS + PA + PC	0.6 ± 0.1 mg	-	0.6 ± 0.1 mg	0.6 ± 0.1 mg	0.6 ± 0.1 mg
PP + PS + PA + PC	-	0.6 ± 0.1 mg	0.6 ± 0.1 mg	0.6 ± 0.1 mg	0.6 ± 0.1 mg
LDPE + PP + PS + PA + PC	0.6 ± 0.1 mg	0.6 ± 0.1 mg	0.6 ± 0.1 mg	0.6 ± 0.1 mg	0.6 ± 0.1 mg

**Table 2:** HDPE, PP and PS weight percentages in extruded blends.

	HDPE [%wt]	PP [%wt]	PS [%wt]
90HDPE-10PP	90	10	-
70HDPE-30PP	70	30	-
50HDPE-50PP	50	50	-
30HDPE-70PP	30	70	-
10HDPE-90PP	10	90	-
90HDPE-10PS	90	-	10
50HDPE-50PS	50	-	50
10HDPE-90PS	10	-	90
90PP-10PS	-	90	10
50PP-50PS	-	50	50
10PP-90PS	-	10	90

**Table 3:** Pyrolyzer, GC and MS parameters.

Pyrolyzer	Equilibration time (s)	5
	End time (min)	5
	Initial temperature (°C)	70
	Delay time (s)	0
	Ramp rate 1 (°C/s)	60
	Hold temperature 1 (°C)	400
	Ramp rate 2 (°C/s)	60
	Hold temperature 2 (°C)	600
	Hold time 2 (s)	231
	Column flow/inlet pressure time 1 (s)	180
GC	Column oven temperature (°C)	70
	Hold time column oven temperature (min)	1
	Injection temperature (°C)	100
	Injection mode	Split
	Pressure (kPa)	62.5
	Total row (mL/min)	84
	Column row (mL/min)	1
	Linear velocity (cm/s)	36.8
	Purge flow (mL/min)	3
	Split ratio	80
	Rate (°C/min)	10
	Final temperature (°C)	300
	Hold time final temperature (min)	4.5
	Total program time (min)	28.5
MS	Ion source temperature (°C)	200
	Interface temperature (°C)	200
	Solvent cut time (min)	0.5
	Start time (min)	1
	End time (min)	28
	Acquisition mode	Scan
	Event time (min)	0.3
	Scan speed	250
	Start *m*/*z*	35
	End *m*/*z*	380

**Table 4:** Pyrolysis products for qualitative identification.

Peak Notation	Characteristic Pyrolysis Product	Retention Time (min)	Coming From
**E1**	1,9-decadiene	6.46	LDPE
**E2**	1-decene	6.61	LDPE
**E3**	Decane	6.75	LDPE
**P1**	2,4-dimethyl-1-heptene	4.74	PP
**S1**	Styrene	5.38	PS
**A1**	ε-caprolactam	10.80	PA
**C1**	2,2-bis(4′-methoxylphenyl)propane	21.2	PC

**Table 5:** Peaks’ detection for several mixtures.

	E1	E2	E3	P1	S1	A1	C1
LDPE + PP	Detected	Detected	Not detected	Detected	-	-	-
LDPE + PS	Detected	Detected	Detected	-	Detected	-	-
PP + PS	-	-	-	Detected	Detected	-	-
PS + PA	-	-	-	-	Detected	Detected	-
PP + PS + PC	-	-	-	Detected	Detected	-	Detected
LDPE + PP + PS	Not detected	Detected	Not detected	Detected	Detected	-	-
LDPE + PA + PC	Not detected	Detected	Detected	-	-	Detected	Detected
LDPE + PS + PA	Not detected	Detected	Detected	-	Detected	Detected	-
LDPE + PS + PC	Not detected	Detected	Detected	-	Detected	-	Detected
LDPE + PP + PS + PA	Not detected	Detected	Not detected	Detected	Detected	Detected	-
LDPE + PP + PA + PC	Not detected	Detected	Not detected	Detected	-	Detected	Detected
LDPE + PP + PS + PC	Not detected	Detected	Not detected	Detected	Detected	-	Detected
LDPE + PS + PA + PC	Not detected	Detected	Detected	-	Detected	Detected	Detected
PP + PS + PA + PC	-	-	-	Detected	Detected	Detected	Detected
LDPE + PP + PS + PA + PC	Not detected	Detected	Not detected	Detected	Detected	Detected	Detected

**Table 6:** Pyrolysis products for quantitative identification.

Characteristic Pyrolysis Product	Retention Time (min)	Coming From
1-decene	6.61	HDPE
1-dodecene	9.47	HDPE
2,4-dimethyl-1-heptene	4.69	PP
1-undecene, 7-methyl-	11.17	PP
Bibenzyl	14.10	PS
2,5-dyphenyl, 1,5-hexadiene	18.29	PS

**Table 7:** R^2^, standard deviation of the intercept and values of slope for HDPE, PP and PS blends.

		Blend	R^2^	Intercept SD	Slope
HDPE	1-decene	HDPE-PP	0.92	2.14 × 10^6^	4.70 × 10^7^
		HDPE-PS	0.87	3.54 × 10^6^	4.70 × 10^7^
	1-dodecene	HDPE-PP	0.89	1.42 × 10^6^	2.69 × 10^7^
		HDPE-PS	0.86	2.10 × 10^6^	2.71 × 10^7^
PP	2,4-dimethyl-1-heptene	HDPE-PP	0.90	6.55 × 10^6^	1.29 × 10^8^
		PP-PS	0.95	6.94 × 10^6^	1.55 × 10^8^
	1-undecene, 7-methyl-	HDPE-PP	0.92	1.87 × 10^6^	4.35 × 10^7^
		PP-PS	0.94	2.24 × 10^6^	4.46 × 10^7^
PS	Bibenzyl	HDPE-PS	0.93	7.24 × 10^5^	1.36 × 10^7^
		PP-PS	0.91	1.11 × 10^6^	1.42 × 10^7^
	2,5-diphenyl-1,5-hexadiene	HDPE-PS	0.88	6.68 × 10^5^	9.60 × 10^6^
		PP-PS	0.98	2.43 × 10^5^	9.32 × 10^6^

**Table 8:** LOD and LOQ for HDPE, PP and PS blends.

		Blend	LOD [mg]	LOD [%]	LOQ [mg]	LOD [%]
HDPE	1-decene	HDPE-PP	0.15	25	0.45	77
		HDPE-PS	0.24	32	0.70	97
	1-dodecene	HDPE-PP	0.17	28	0.52	88
		HDPE-PS	0.26	33	0.79	-
PP	2,4-dimethyl-1-heptene	HDPE-PP	0.17	28	0.52	85
		PP-PS	0.15	25	0.45	75
	1-undecene, 7-methyl-	HDPE-PP	0.14	23	0.42	72
		PP-PS	0.17	28	0.52	83
PS	Bibenzyl	HDPE-PS	0.18	25	0.55	77
		PP-PS	0.26	42	0.79	-
	2,5-diphenyl-1,5-hexadiene	HDPE-PS	0.23	38	0.70	-
		PP-PS	0.09	15	0.27	43

## Data Availability

Data are available upon request

## Supplementary Materials

The following supporting information can be downloaded at: https://www.mdpi.com/article/10.3390/polym16010071/s1, Figure S1. LDPE chromatogram; Figure S2. PP chromatogram; Figure S3. PS chromatogram; Figure S4. PA chromatogram; Figure S5. PC chromatogram; Figure S6. LDPE + PS chromatogram; Figure S7. LDPE + PS chromatogram comparison; Figure S8. PP + PS chromatogram; Figure S9. PP + PS chromatogram comparison; Figure S10. PS + PA chromatogram; Figure S11. PS + PA chromatogram comparison; Figure S12. LDPE + PP + PS chromatogram; Figure S13. LDPE + PP + PS chromatogram comparison; Figure S14. PP + PS + PC chromatogram; Figure S15. PP + PS + PC chromatogram comparison; Figure S16. LDPE + PA + PC chromatogram; Figure S17. LDPE + PA + PC chromatogram comparison; Figure S18. LDPE + PS + PA chromatogram; Figure S19. LDPE + PS + PA chromatogram comparison; Figure S20. LDPE + PS + PC chromatogram; Figure S21. LDPE + PS + PC chromatogram comparison; Figure S22. LDPE + PP + PS + PA chromatogram; Figure S23. LDPE + PP + PS + PA chromatogram comparison; Figure S24. LDPE + PP + PA + PC chromatogram; Figure S25. LDPE + PP + PA + PC chromatogram comparison; Figure S26. LDPE + PP + PS + PC chromatogram; Figure S27. LDPE + PP + PS + PC chromatogram comparison; Figure S28. LDPE + PS + PA + PC chromatogram; Figure S29. LDPE + PS + PA + PC chromatogram comparison. Table S1: Peaks areas of HDPE and PP in HDPE-PP blends; Table S2. Peaks areas of HDPE and PS in HDPE-PS blends; Table S3. Peaks areas of PP and PS in PP-PS blends.
