# Supplementary material for: Identification of Plastics in Mixtures and Blends through Pyrolysis-Gas Chromatography/Mass Spectrometry
Source: Polymers (Basel). 2023 Dec 26;16(1):71. doi: 10.3390/polym16010071 (PMC10780318; doi:10.3390/polym16010071)
Supplement: Supplementary file 1 [file polymers-16-00071-s001.zip › polymers-2755018-supplementary.pdf]

Supplementary information

Figure S1, Figure S2, Figure S3, Figure S4 and Figure S5 show the chromatograms of each polymer.

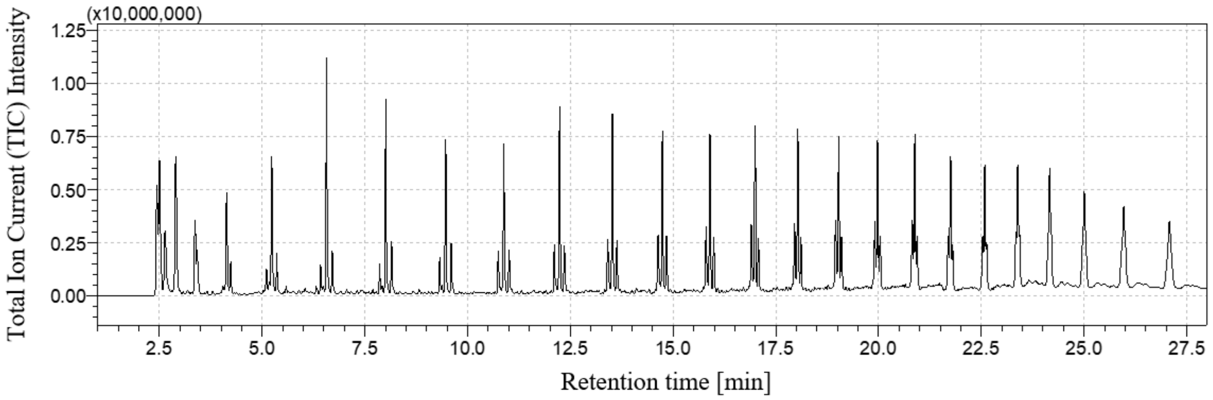

Figure S1. LDPE chromatogram.

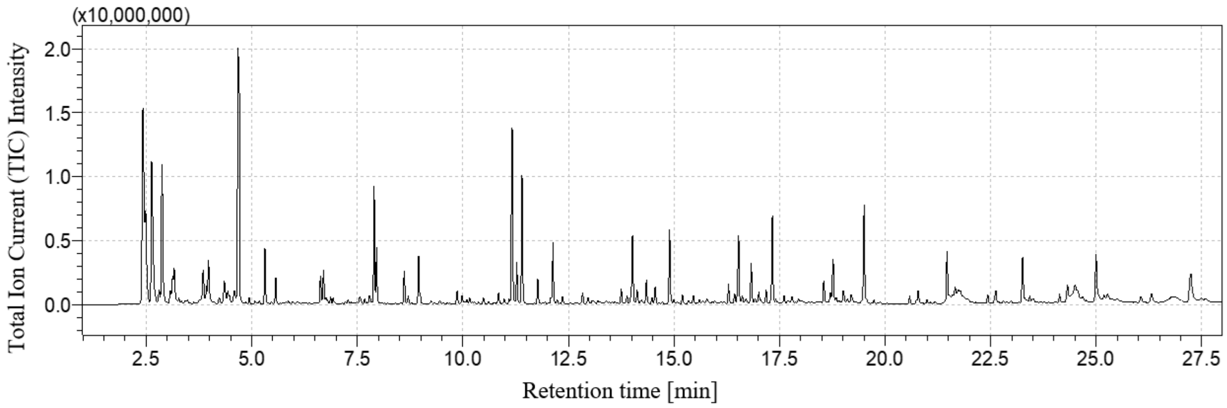

Figure S2. PP chromatogram.

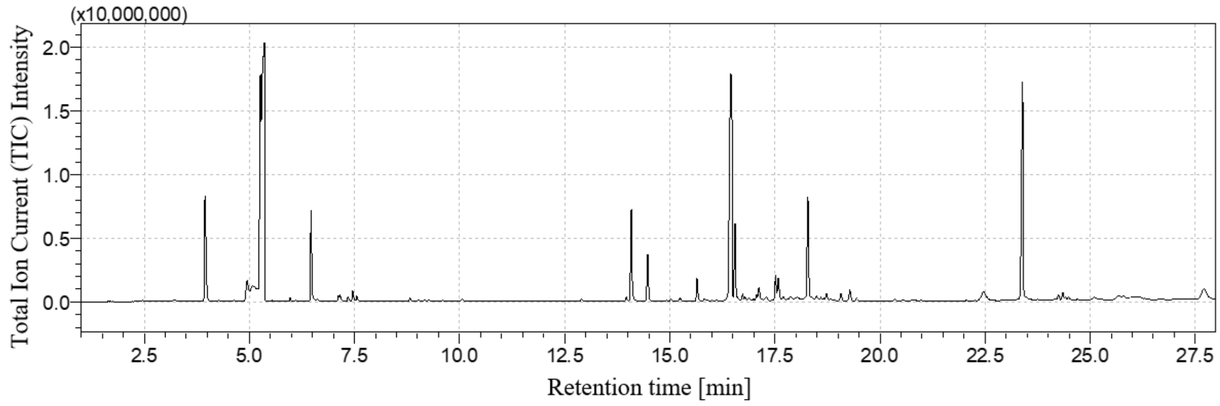

Figure S3. PS chromatogram.

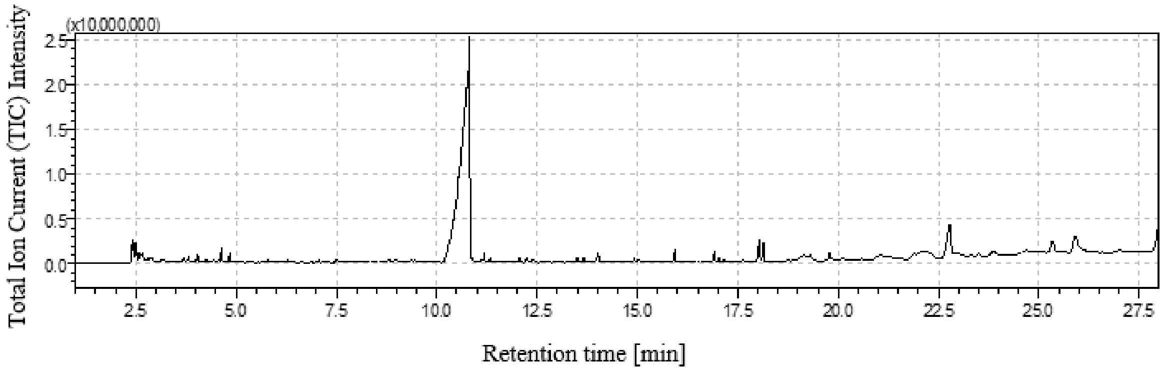

Figure S4. PA chromatogram.

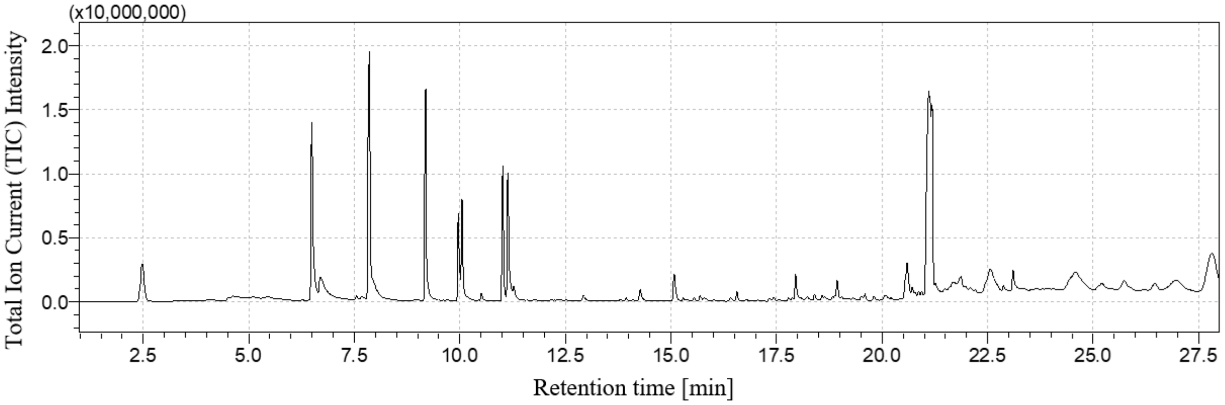

Figure S5. PC chromatogram.

Figure S6 and Figure S7 show LDPE+PS chromatogram and its comparison with LDPE and PS chromatograms.

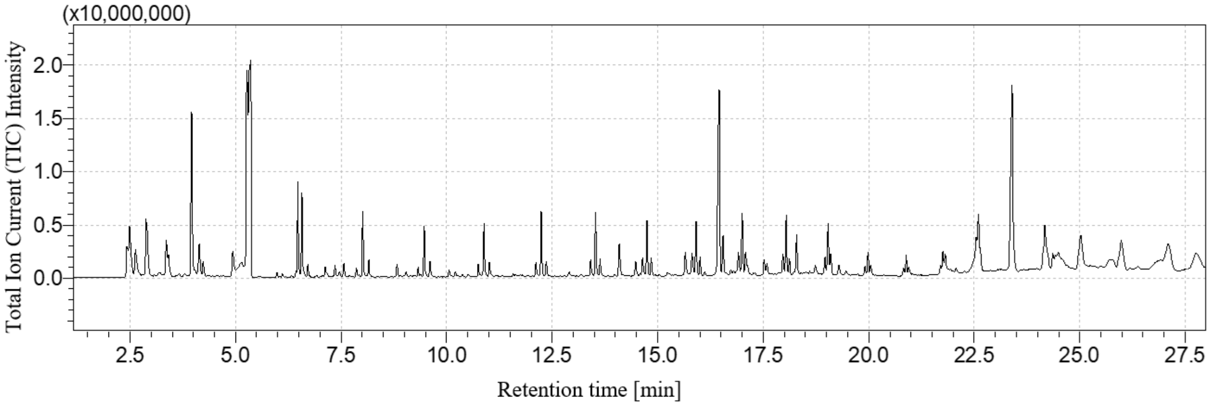

Figure S6. LDPE+PS chromatogram.

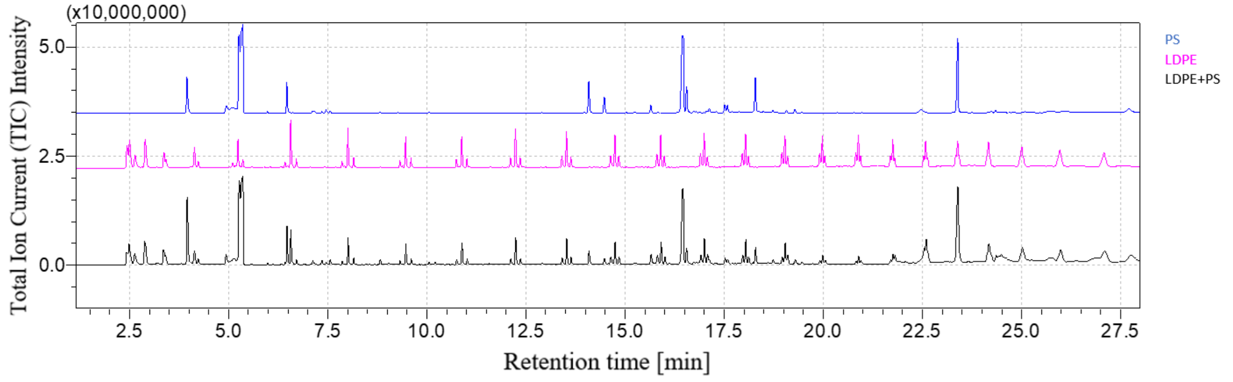

Figure S7. LDPE+PS chromatogram comparison.

Figure S8 and Figure S9 show PP+PS chromatogram and its comparison with PP and PS chromatograms.

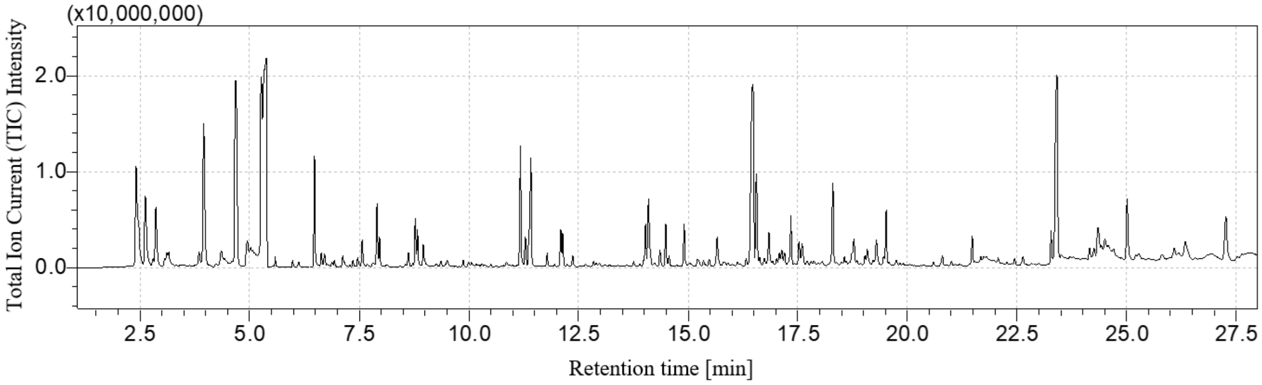

Figure S8. PP+PS chromatogram.

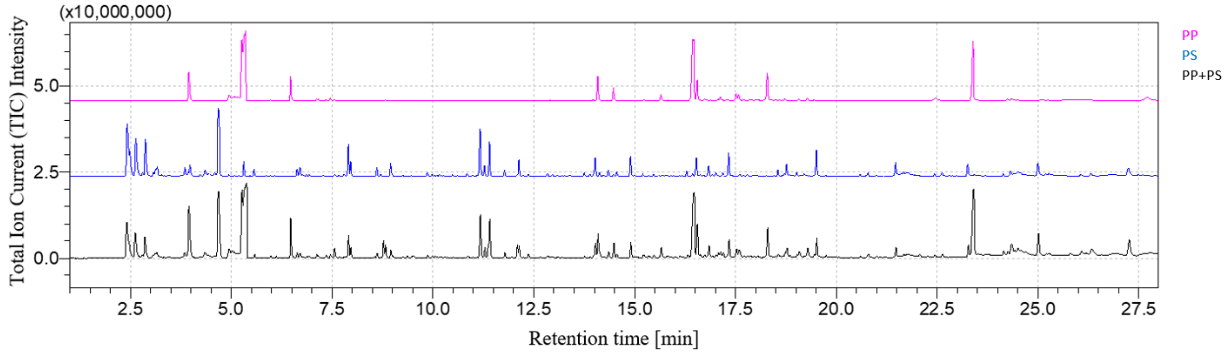

Figure S9. PP+PS chromatogram comparison.

Figure S10 and Figure S11 show PS+PA chromatogram and its comparison with PA and PS chromatograms.

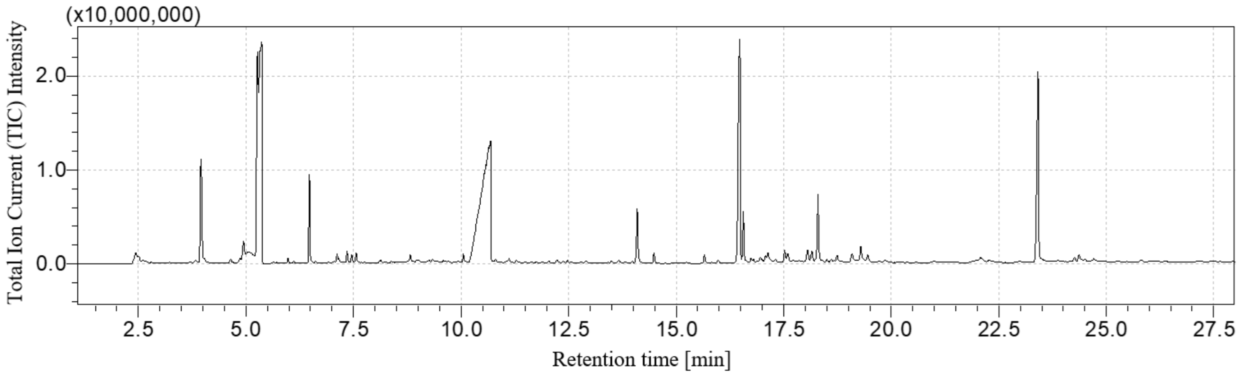

Figure S10. PS+PA chromatogram.

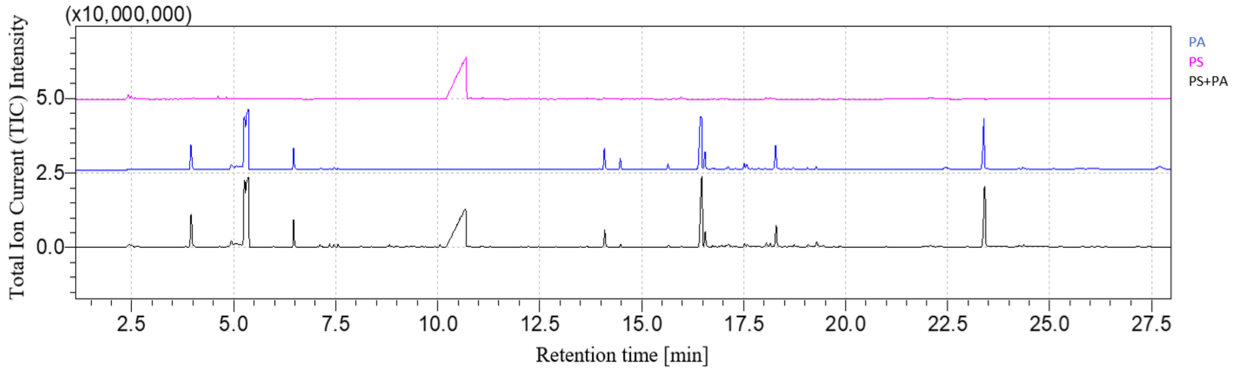

Figure S11. PS+PA chromatogram comparison.

Figure S12 and

Figure **S13** show LDPE+PP+PS chromatogram and its comparison with LDPE, PP and PS chromatograms.

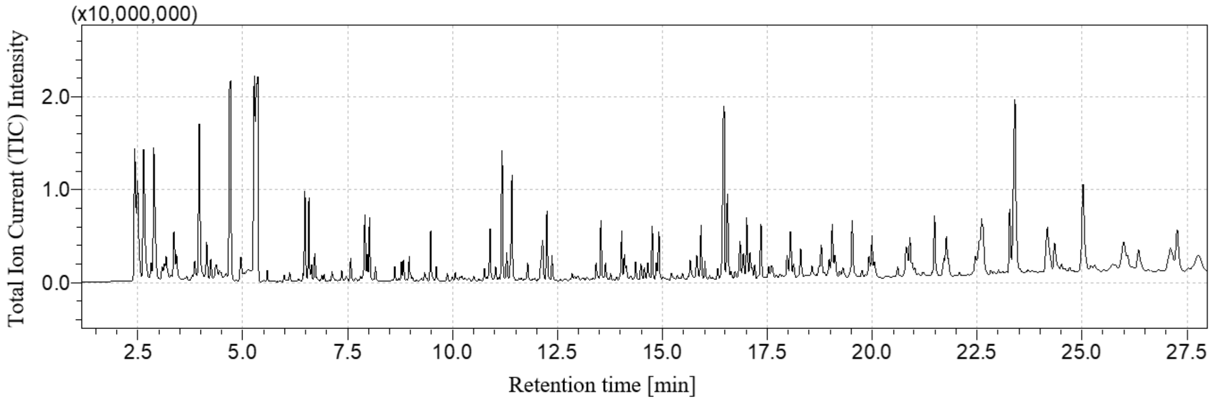

Figure S12. LDPE+PP+PS chromatogram.

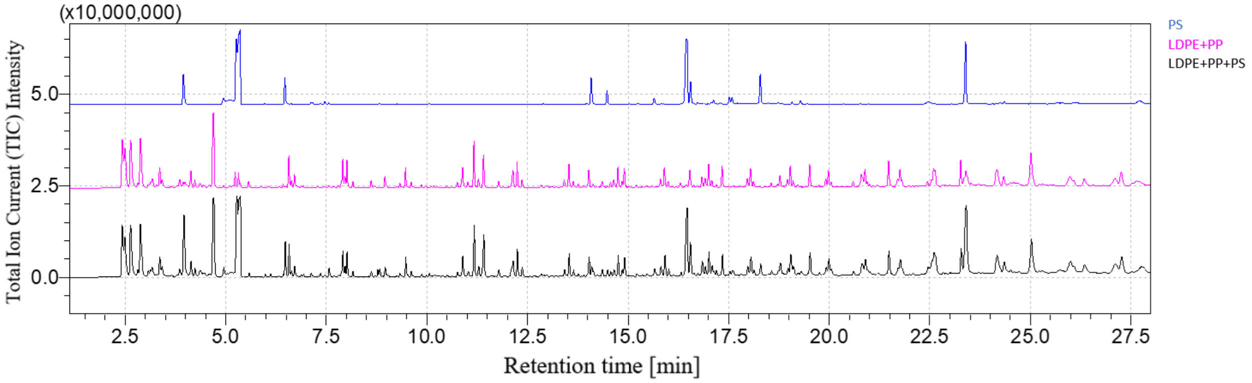

Figure S13. LDPE+PP+PS chromatogram comparison.

Figure S14 and Figure S15 show PP+PS+PC chromatogram and its comparison with PP, PS and PC chromatograms.

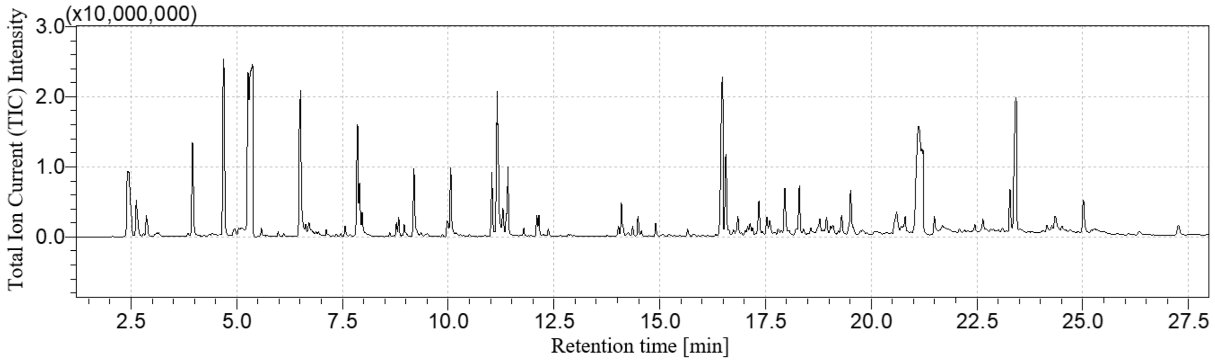

Figure S14. PP+PS+PC chromatogram.

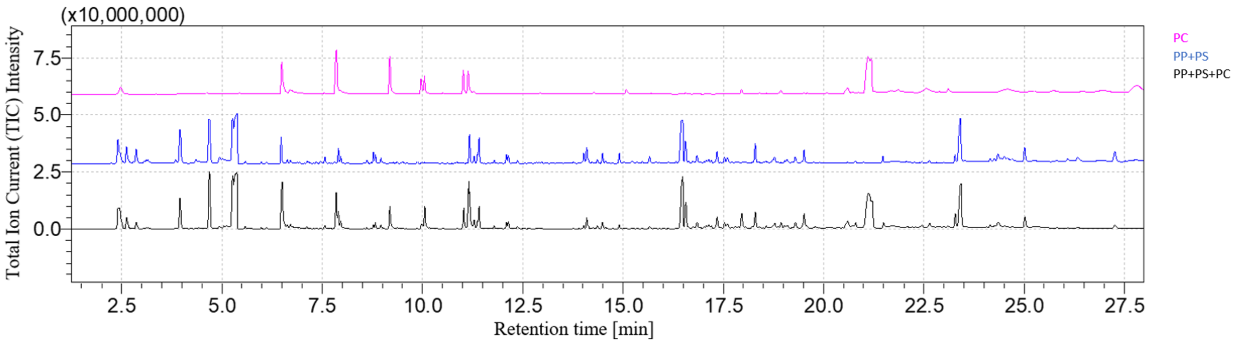

Figure S15. PP+PS+PC chromatogram comparison.

Figure S16 and

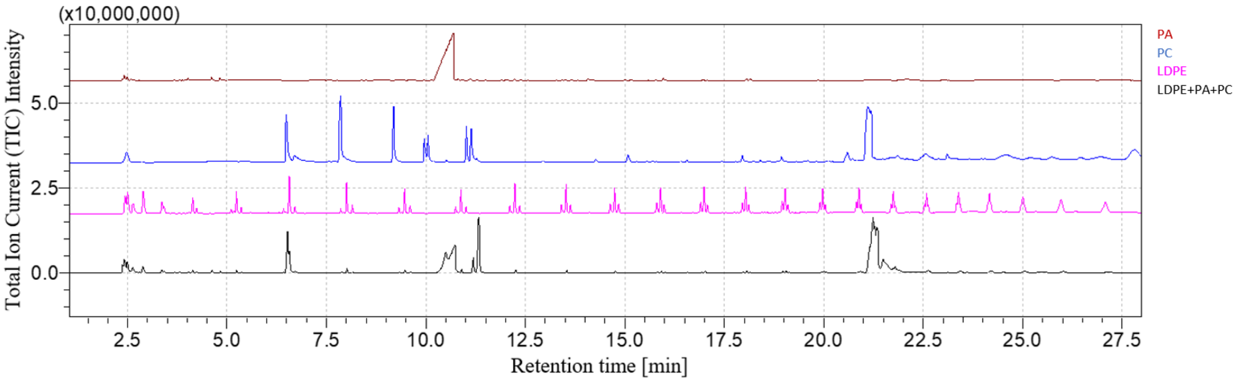

Figure **S17** show LDPE+PA+PC chromatogram and its comparison with LDPE, PA and PC chromatograms.

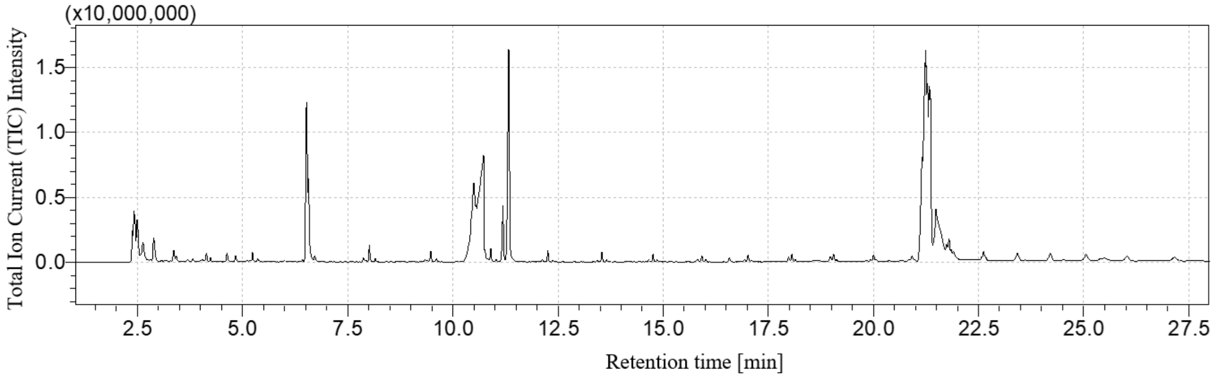

Figure S16. LDPE+PA+PC chromatogram.

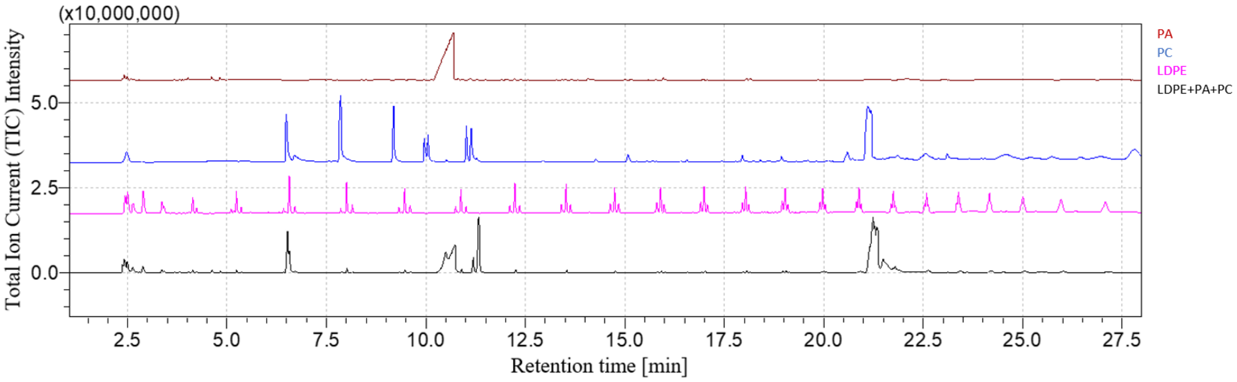

Figure S17. LDPE+PA+PC chromatogram comparison.

Figure S18 and Figure S19 show LDPE+PS+PA chromatogram and its comparison with LDPE, PS and PA chromatograms.

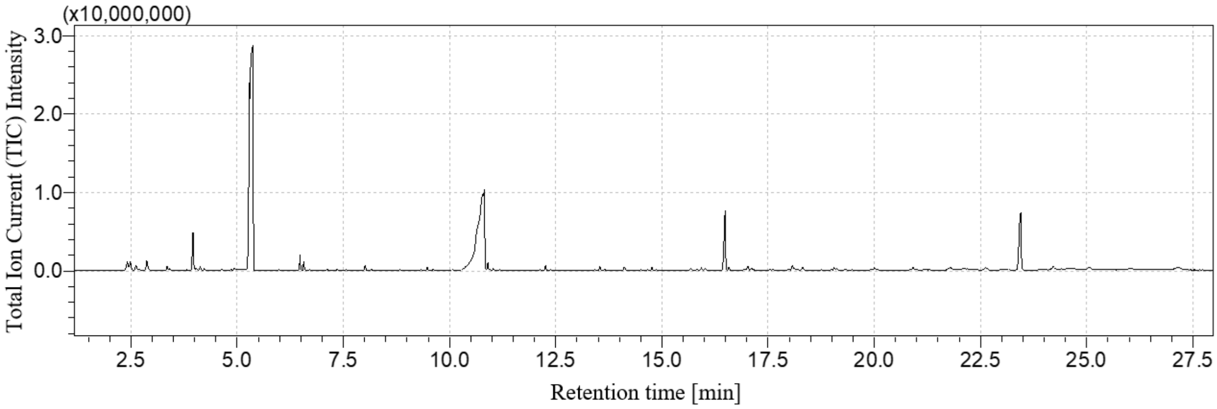

Figure S18. LDPE+PS+PA chromatogram.

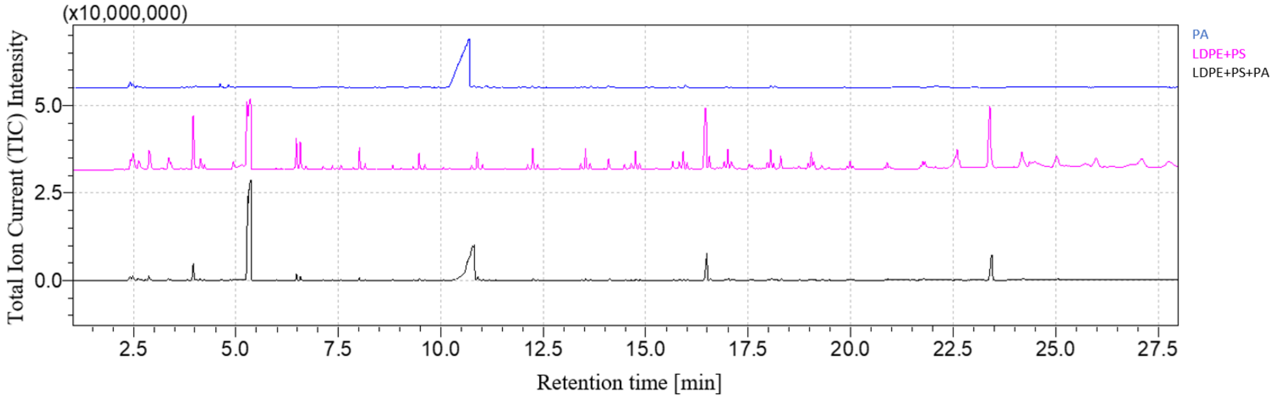

Figure S19. LDPE+PS+PA chromatogram comparison.

Figure S20 and Figure S21 show LDPE+PS+PC chromatogram and its comparison with LDPE, PS and PC chromatograms.

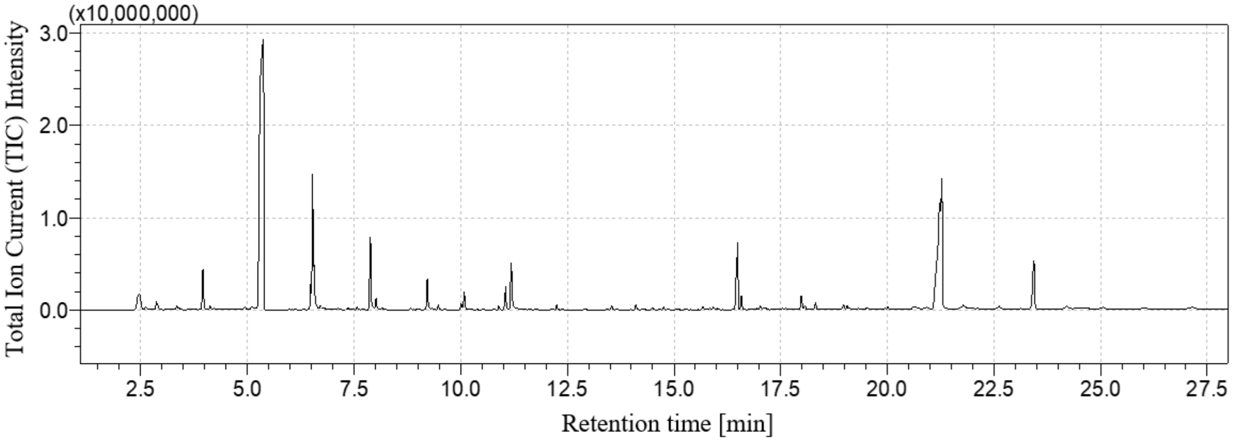

Figure S20. LDPE+PS+PC chromatogram.

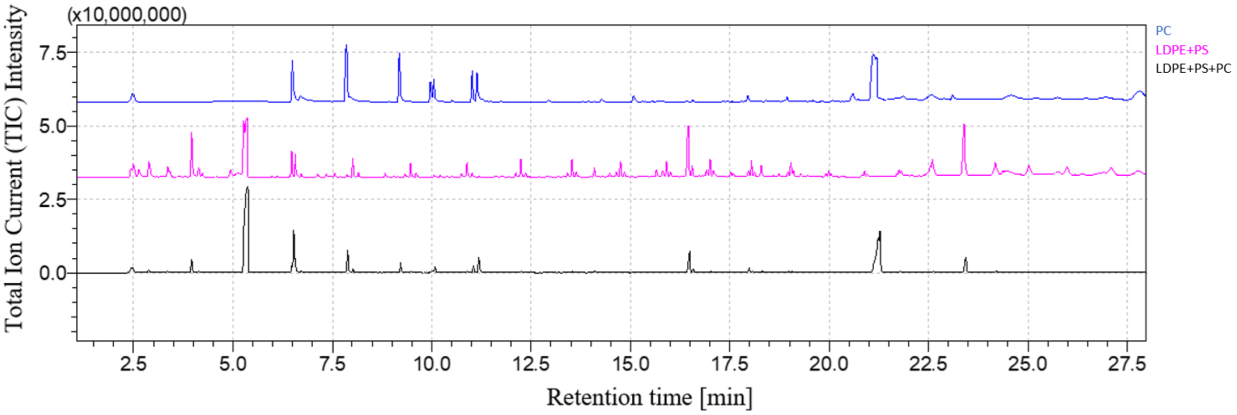

Figure S21. LDPE+PS+PC chromatogram comparison.

Figure S22 and Figure S23 show LDPE+PP+PS+PA chromatogram and its comparison with LDPE+PP and PS+PA chromatograms.

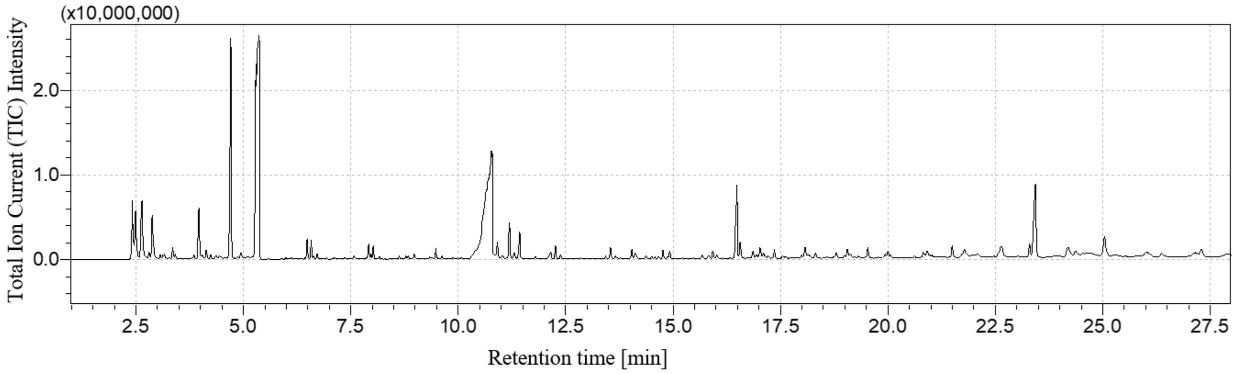

Figure S22. LDPE+PP+PS+PA chromatogram.

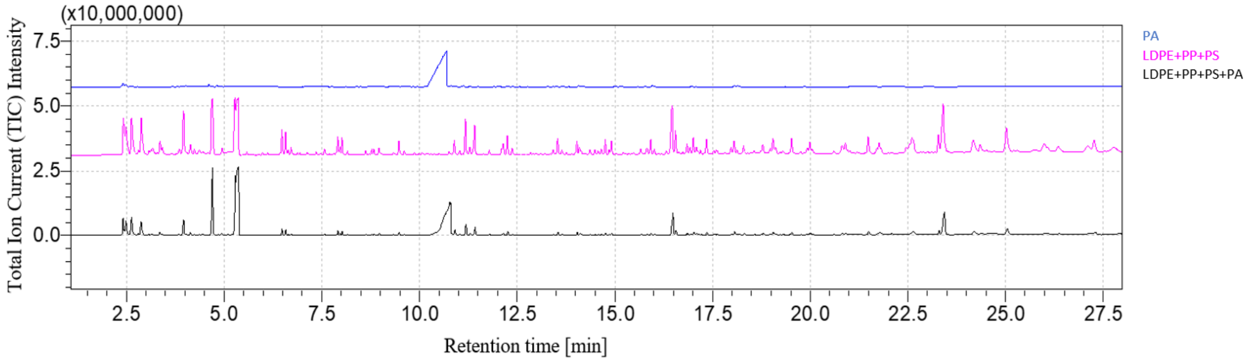

Figure S23. LDPE+PP+PS+PA chromatogram comparison.

Figure S24 and Figure S25 show LDPE+PP+PA+PC chromatogram and its comparison with LDPE+PA+PC and PP chromatograms.

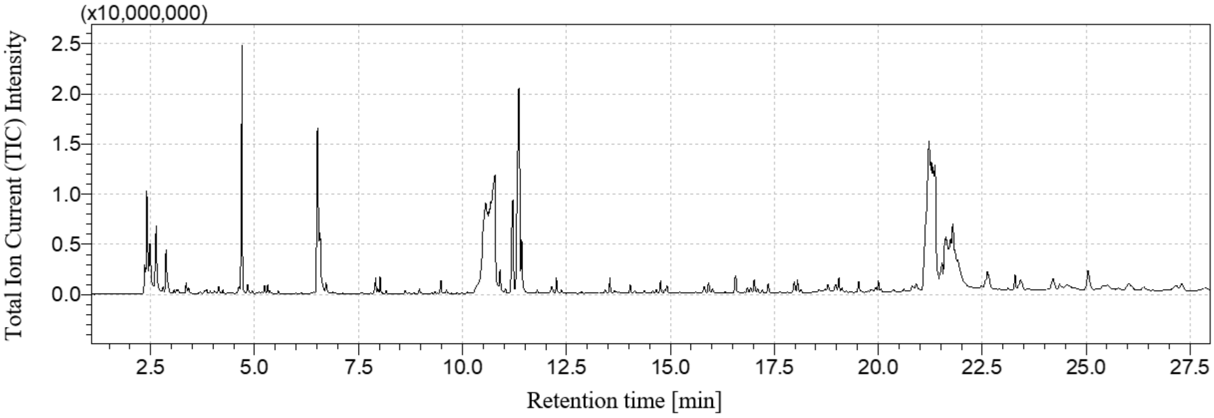

Figure S24. LDPE+PP+PA+PC chromatogram.

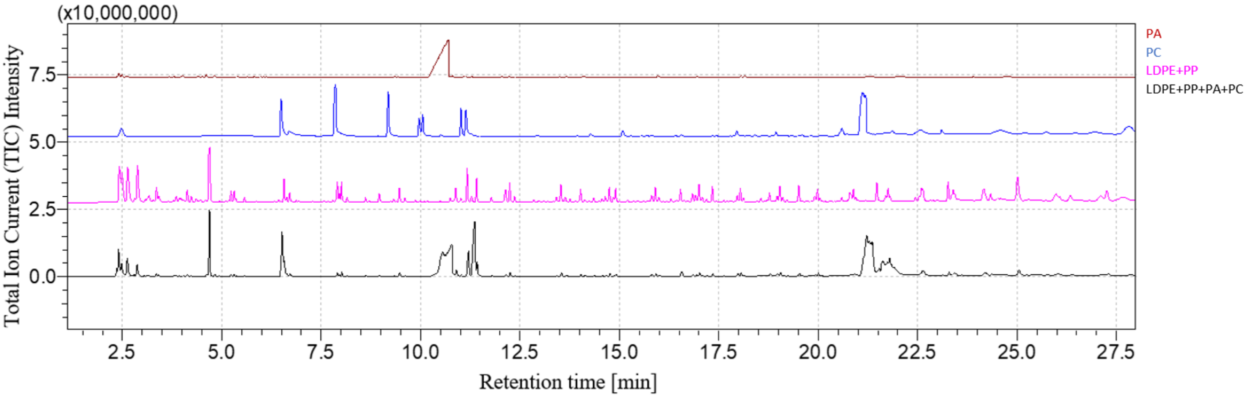

Figure S25. LDPE+PP+PA+PC chromatogram comparison.

Figure S26 and

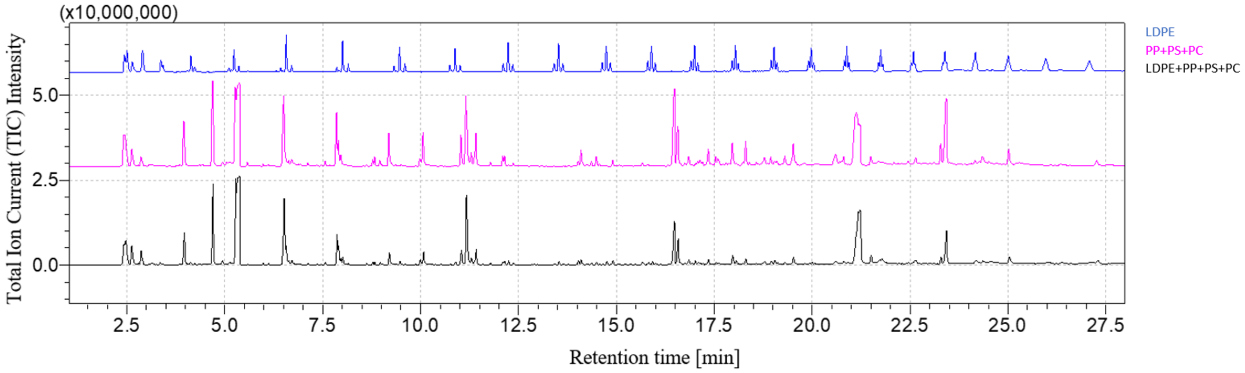

Figure **S27** show LDPE+PP+PS+PC chromatogram and its comparison with PP+PS+PC and LDPE chromatograms.

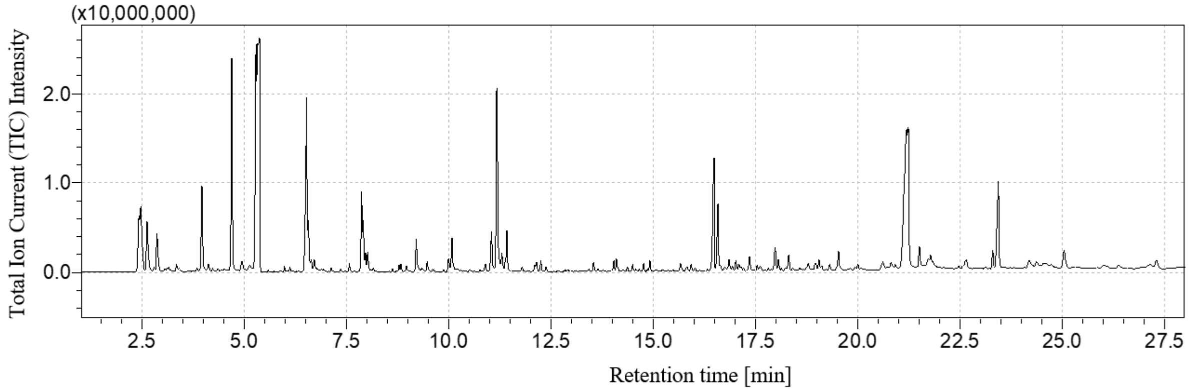

Figure S26. LDPE+PP+PS+PC chromatogram.

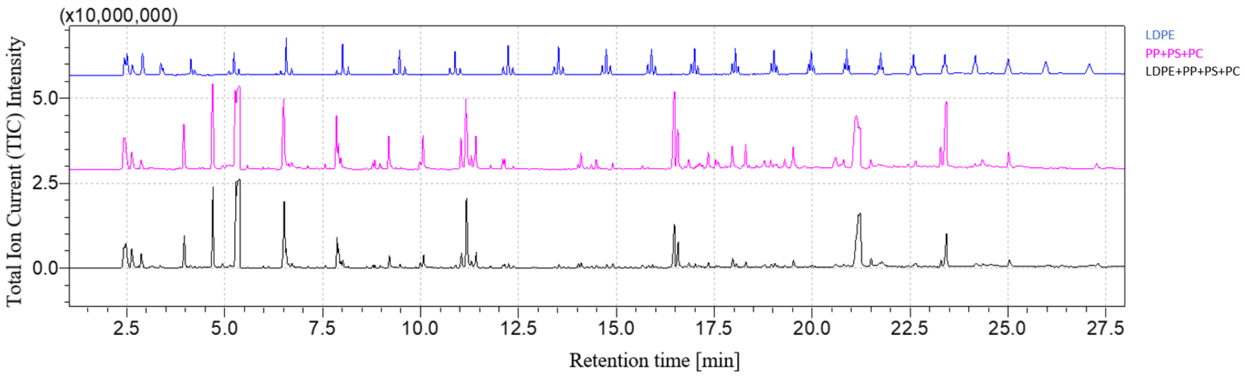

Figure S27. LDPE+PP+PS+PC chromatogram comparison.

Figure S28 and Figure S29 show LDPE+PS+PA+PC chromatogram and its comparison with LDPE+PS+PC and PA chromatograms.

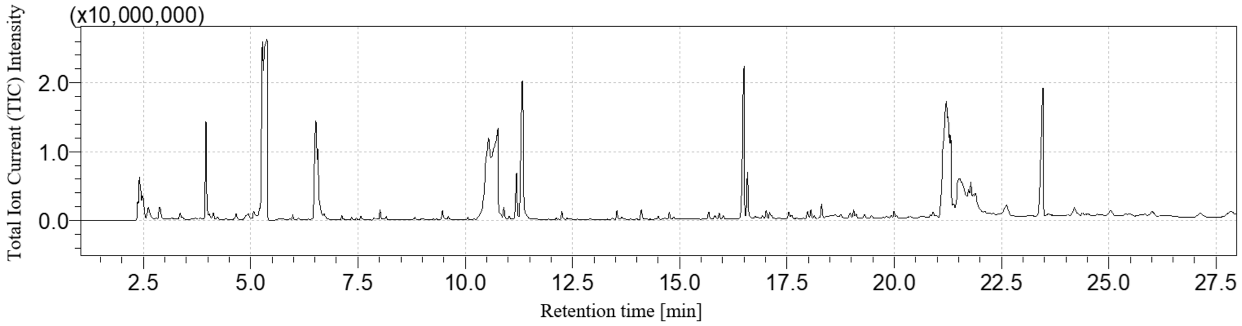

Figure S28. LDPE+PS+PA+PC chromatogram.

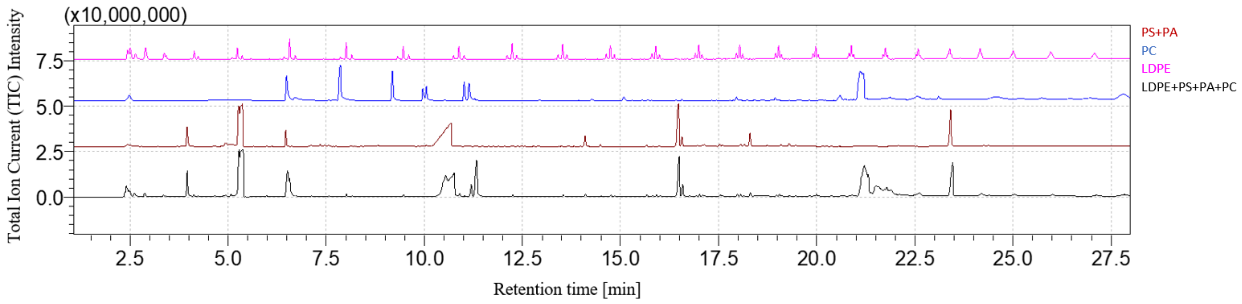

Figure S29. LDPE+PS+PA+PC chromatogram comparison.

Table S1, Table S2 and Table S3 report the values of peaks areas of the characteristic markers of HDPE, PP and PS in different types of blends.

HDPE [%wt]	HDPE [mg]	PP [%wt]	PP [mg]	1-decene area [TIC]	1-dodecene area [TIC]	2,4-dimethyl-1-heptene [TIC]	1-undecene, 7-methyl [TIC]
10	0.06	90	0.54	2.75E6	1.34E6	8.05E7	2.44E7
30	0.18	70	0.42	6.16E6	3.34E6	7.96E7	1.78E7
50	0.30	50	0.30	8.83E6	4.47E6	4.90E7	6.63E6
70	0.42	30	0.18	1.67E7	9.03E6	4.50E7	5.43E6
90	0.54	10	0.06	2.11E7	1.17E7	2.53E7	3.87E6
100	0.6	0	0	3.18E7	1.84E7	0	0
0	0	100	0.6	0	0	8.16E7	8.16E7

Table S1. Peaks areas of HDPE and PP in HDPE-PP blends.

HDPE [%wt]	HDPE [mg]	PS [%wt]	PS [mg]	1-decene area [TIC]	1-dodecene area [TIC]	Bibenzyl area [TIC]	2,5-diphenyl-1,5-hexadiene [TIC]
10	0.06	90	0.54	604693	377339	6.33E6	5.62E6
50	0.30	50	0.30	8.38E6	4.49E6	2.24E6	4.61E6
90	0.54	10	0.06	1.87E7	1.07E7	309195	568575
100	0.6	0	0	3.18E7	1.84E7	0	0
0	0	100	0.6	0	0	8.58E6	5.40E6

Table S2. Peaks areas of HDPE and PS in HDPE-PS blends.

PP [%wt]	PP [mg]	PS [%wt]	PS [mg]	2,4-dimethyl-1-heptene area [TIC]	1-undecene, 7-methyl [TIC]	Bibenzyl area [TIC]	2,5-diphenyl-1,5-hexadiene [TIC]
100	0.6	0	0	8.16E7	2.55E7	0	0
90	0.54	10	0.06	9.08E7	2.41E7	-	363284
50	0.3	50	0.3	3.32E7	6.79E6	2.18E6	2.10E6
10	0.06	90	0.54	2.30E6	1.02E6	6.94E6	5.08E6
0	0	100	0.6	0	0	8.58E6	5.40E6

Table S3. Peaks areas of PP and PS in PP-PS blends.
